# Amniotic Fluid Deficiency and Congenital Abnormalities both Influence Fluctuating Asymmetry in Developing Limbs of Human Deceased Fetuses

**DOI:** 10.1371/journal.pone.0081824

**Published:** 2013-11-27

**Authors:** Clara Mariquita Antoinette ten Broek, Jessica Bots, Irma Varela-Lasheras, Marianna Bugiani, Frietson Galis, Stefan Van Dongen

**Affiliations:** 1 Group of Evolutionary Ecology, University of Antwerp, Antwerp, Belgium; 2 Department of Patterning and Morphogenesis, Gulbenkian Institute of Science, Oeiros, Portugal; 3 Department of Pathology, VU Medical Centre, Amsterdam, The Netherlands; 4 Department of Terrestrial Zoology, Naturalis Biodiversity Center, Leiden, The Netherlands; Tabriz University of Medical Sciences, Iran (Islamic Republic Of)

## Abstract

Fluctuating asymmetry (FA), as an indirect measure of developmental instability (DI), has been intensively studied for associations with stress and fitness. Patterns, however, appear heterogeneous and the underlying causes remain largely unknown. One aspect that has received relatively little attention in the literature is the consequence of direct mechanical effects on asymmetries. The crucial prerequisite for FA to reflect DI is that environmental conditions on both sides should be identical. This condition may be violated during early human development if amniotic fluid volume is deficient, as the resulting mechanical pressures may increase asymmetries. Indeed, we showed that limb bones of deceased human fetuses exhibited increased asymmetry, when there was not sufficient amniotic fluid (and, thus, space) in the uterine cavity. As amniotic fluid deficiency is known to cause substantial asymmetries and abnormal limb development, these subtle asymmetries are probably at least in part caused by the mechanical pressures. On the other hand, deficiencies in amniotic fluid volume are known to be associated with other congenital abnormalities that may disturb DI. More specifically, urogenital abnormalities can directly affect/reduce amniotic fluid volume. We disentangled the direct mechanical effects on FA from the indirect effects of urogenital abnormalities, the latter presumably representing DI. We discovered that both factors contributed significantly to the increase in FA. However, the direct mechanical effect of uterine pressure, albeit statistically significant, appeared less important than the effects of urogenital abnormalities, with an effect size only two-third as large. We, thus, conclude that correcting for the relevant direct factors allowed for a representative test of the association between DI and stress, and confirmed that fetuses form a suitable model system to increase our understanding in patterns of FA and symmetry development.

## Introduction

The bilateral symmetrical traits are determined by the same genes and are replicas of the same developmental event on either side of the body. Under ideal conditions they should develop into identical but mirrored phenotypes. However, small developmental perturbations may accumulate separately on both sides, causing small directionally random asymmetries termed fluctuating asymmetry[1]. Fluctuating asymmetry (FA) and is considered to be a measure of developmental instability (DI), reflecting the inability of an individual to buffer its development against developmental noise [1-6]. Increased levels of environmental or genetic perturbations can cause a breakdown in this buffering capacity herewith increasing DI and FA. Over the past half century a great number of publications have accumulated on the role of FA as measure of DI. However, results in the literature are very heterogeneous and the proximate causes of this diversity remain poorly understood [1]. Despite the extensive body of literature on FA and DI and the recent attempts to explain the heterogeneity in the literature, little attention has been paid to the biologically relevant mechanisms that may influence the relationship between FA and stress/quality. Recently, it has been recognized that development of bilateral traits, such as limbs or eyes, may not experience identical environmental conditions on left and right side to begin with [7-9], which blurs the link between FA and DI and complicates the interpretation of patterns in asymmetry. For example, the levels of FA can be confounded by lateralized behavior when differential use of left and right limbs causes a difference in mechanical loading and influences asymmetry directly (instead of an accumulation of developmental noise when DI induces asymmetry) [7,8]. Also, fetal limb bones have been reported to show directionally asymmetrical development *in utero*, but results are mixed [10-14], (and unpublished manuscript Van Dongen et. al.). Since mechanical loading experienced *in utero* is usually small due to the presence of amniotic fluid, mammalian fetuses have been invoked as interesting model systems to study FA [15]. However, it has been recognized in clinical studies that the (symmetrical) development of limb bones in fetuses can be severely compromised when the amniotic cavity becomes suboptimal for bone growth and remodeling (e.g.[16-18] and see 19 for a recent review). This situation may thus be directly causing asymmetry, instead of the asymmetry (i.e. FA) emerging as accumulation of small developmental errors. 

**Figure 1:**
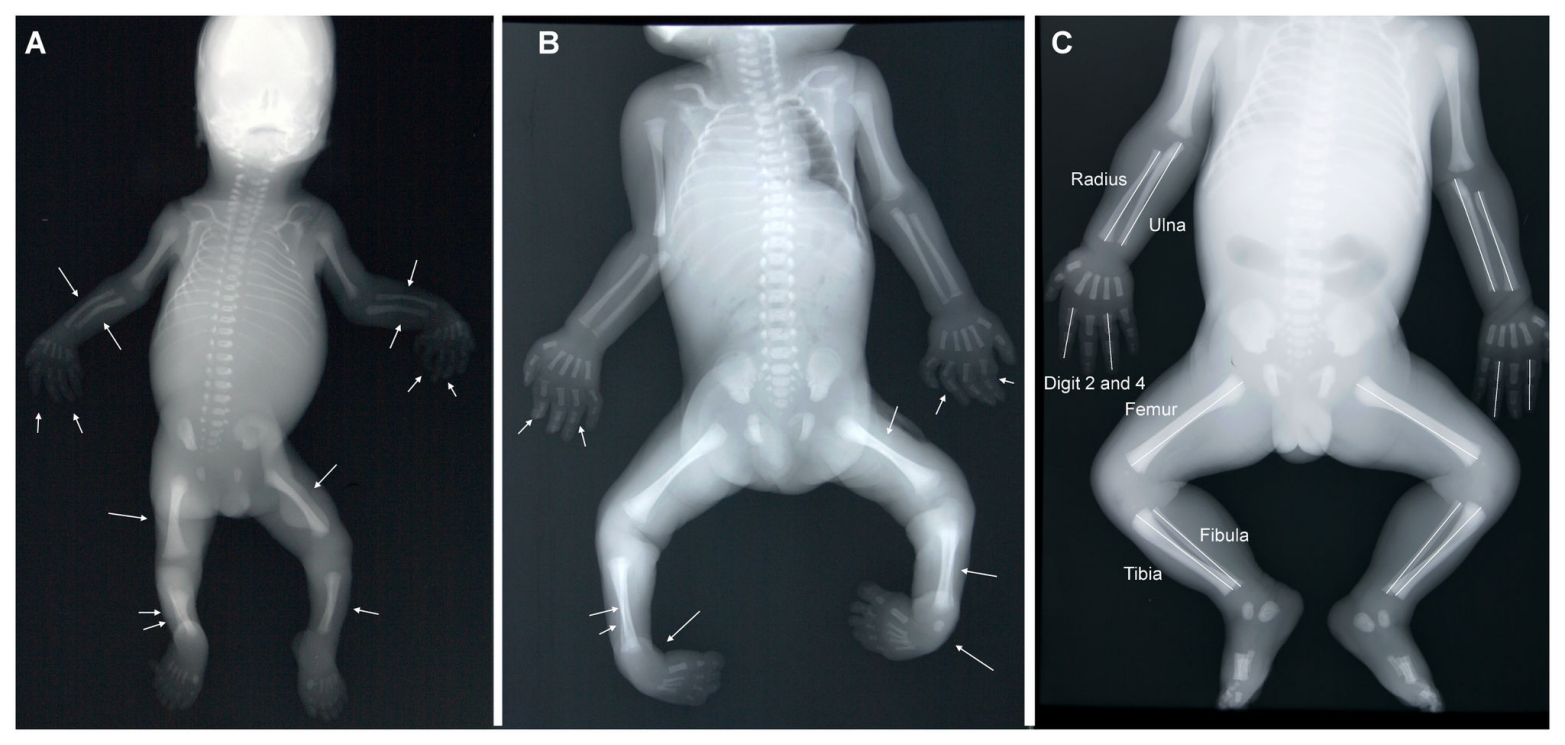
Examples of fetuses that should be excluded and included from FA measurements. A: Fetus with limb deformities (indicated by arrows), which include bowing of the radius and ulna, bowing of the digits, bowing and hypoplasia of the femur, tibia and fibula. B: Fetus with limb deformities including bowing of the digits, bowing and hypoplasia of the femur, tibia and fibula, and talipes equinovarus (clubfeet). The deformed traits in fetus A and B should be excluded from FA measurements. C: Non-deformed, representative fetus, that allows FA measurements: the limbs are properly positioned and no deformities are present. Here, fetuses A and B developed with reduced amniotic fluid volumes and fetus C developed with a normal amniotic fluid volume.

The amniotic fluid in the amniotic cavity has numerous important (mechanical) functions, probably the most significant one of them being the expansion of the amniotic sac. Unimpeded morphogenesis, especially from the fetal extremities, is only possible when there is no mechanical constraint for growth of the uterine cavity [17-20]. Moreover, amniotic fluid protects the fetus against injuries from external trauma [21] and enlargement of the intrauterine space by expansion of the amniotic cavity is a prerequisite for fetal viability as expansion of the thoracic cavity allows adequate lung development [16,22]. The absence (AH, anhydramnios) or deficiency (OH, oligohydramnios) of amniotic fluid may compromise normal symmetrical development of the limb bones via growth constraints and cause deformations or induce the development of severe malformations (e.g., Figure 1A,B). Hence, when the asymmetry emerges more subtly and, therefore potentially unnoticed, it may confound the use of FA as a measure of DI. However, such mechanical effects during early development and their potential confounding effects through associations with other measures of stress have not been investigated previously. For example, Van Dongen et al. [23] found four different groups of congenital abnormalities to increase FA in fetal limb bones. Yet, some of these abnormalities are found associated with AH/OH in the clinical literature. Namely, urogenital malformations can cause AH or OH and limb defects and bronchopulmonary abnormalities can be caused directly by AH or OH [18-20,24]. Thus, the observed increase in FA in fetuses with these abnormalities may also be due to direct mechanical pressure caused by AH/OH and not to the accumulation of random developmental noise, i.e., DI [23]. More generally, the relative importance of potentially direct mechanical vs. indirect effects is crucial to evaluate the usefulness of model systems in FA studies and requires a more systematic approach. 

In this study, we attempt to disentangle the direct effects of AH/OH on FA from the indirect effects of the associated congenital abnormalities with AH/OH. To do so, we used a large dataset of deceased human fetuses of which a substantial part did not show directional asymmetry in a previous study with less individuals included in the study [14]. We 1) test the relationship between FA and AH/OH to see whether asymmetry is increased in fetuses who suffered from AH/OH; 2) investigate the relationship between the amounts of amniotic fluid (AH, OH and PH, polyhydramnios: excess of amniotic fluid) and the occurrence of different types of congenital abnormalities; and 3) study the relationship between FA and congenital abnormalities associated with AH/OH. We hypothesize that if direct effects from mechanical pressure contribute to (subtle) asymmetrical development of the limb bones, asymmetry will increase significantly with AH/OH. The relative importance of this direct effect on the strength of associations between FA and congenital abnormalities associated with AH/OH/PH is evaluated by comparing effect sizes when both factors are included in the same model. If FA is increased through DI, we predict that effect sizes are significant for congenital abnormalities associated with FA, even when correcting for the presence of AH/OH. However, if the direct mechanical loading causes the increase in asymmetry, we expect no effects for the congenital abnormalities associated with FA, but positive effect sizes for the presence of AH/OH. When both direct and indirect factors influence levels of asymmetry, we expect positive effect sizes for AH/OH and for the congenital abnormalities associated with AH/OH, when included in the same model. 

The tests thus allowed us to evaluate that direct effects of reduced amniotic fluid volume on subtle asymmetries in limb development exist, but that their influence on FA is only limited compared to the effects of urogenital abnormalities on limb FA. Correcting for the relevant direct factors allowed representative testing of associations between DI and stress. 

## Materials and Methods

### Fetuses and asymmetry measurements

Since 1980, all deceased fetuses and infants presented for medical examination at the VU Medical Centre have been routinely radiographed both ventrally and laterally (23mA, 70–90 kV, 4–12 sec, Agfa [Mortsel, Belgium] Gevaert D7DW Structurix ﬁlms).

This research was carried out on anterior-posterior projections of 1339 deceased fetuses and infants obtained between 1990 and 2009. We excluded all measurements from fetuses that had abnormally developed limbs, because otherwise the possibly higher measured FA could have been an artifact of the abnormalities directly (Figure 1A,B). Further, we excluded fetuses from analyses when limb bones were not properly positioned or radiographs had insufficient resolution. In total, we measured 616 male and 487 female fetuses and infants and 4 fetuses of unknown sex (13.6 - 92.1 weeks, mean: 27.8 weeks + 10.0 weeks) for the length of the left and right radius, ulna, femur, tibia, fibula, digit 2, and digit 4 (Figure 1C) in Image J version 1.42q. To allow for digital image analysis, the radiographs were digitized using a Canon 30D digital camera in a fixed-distance set-up with a glass plate and a flash underneath. Measurements on the digitized radiographs were taken from the midpoint of the proximal end of the bone to the midpoint of the distal end of the bone (Figure 1C). For the measurements of the digits we considered the length of the three phalangeal bones together, i.e., from the proximal end of the proximal phalanx tot the distal end of the distal phalanx (Figure 1C). Standard autopsy reports were filed in a national pathological archive (PALGA; www.palga.nl), where we searched the reports for congenital abnormalities of different organ systems and conditions of the environment of the developing fetus. We categorized the different congenital abnormalities into separate groups of malformations in the different organ systems: bronchopulmonary malformations (BP), craniofacial malformations (CF), cardiovascular malformations (CV), digestive system malformations (DS), limb defects (LD), muscular system defects (MUS), nervous system defects (NS), skeletal malformations (SK), urogenital malformations (UG), and ventral body wall defects (VBW). Not all individuals were in good condition due to maceration, resulting in missing data for some of their organ systems. When autopsy was not approved by the parents, internal organ systems were also scored as missing. 

Where possible, we scored malformations only in one category, by distinguishing between primary and secondary causes, with only primary causes counted in the analyses (see also 25 for further details).

### Ethics Statement

Patient data and radiographs were used according the guidelines of the Medical Ethics Committee of the VU University Medical Center and patient anonymity was strictly maintained. Parental written informed consent was obtained for all patients and data were handled in a coded and completely anonymous fashion, according to Dutch national ethical guidelines (Code for Proper Secondary Use of Human Data, Dutch Federation of Medical Scientific Societies; http://www.federa.org/codes-conduct). 

### Fluctuating asymmetry measurement and the estimation of measurement error and directional asymmetry

Measurements were conducted by four different investigators (JB, CtB, FG and IVL) who had no prior knowledge of the autopsy. Thirty-one fetuses were re-measured independently by each investigator to ensure the accuracy of the measurements. Correlation tests showed that results were highly comparable (*P* < 0.001). In addition, the whole procedure of positioning the fetus and making the radiograph was repeated for 147 individuals. To determine measurement error (ME) and directional asymmetry (DA), we used a mixed regression model [26]. Three types of measurement error could be determined: first on the individual level when repeated measurements were taken on the same individual, second as a result of positioning the fetus based on repeated radiographs of the same individual, and third by measuring different digitized photographs of the same radiograph. To test for directional asymmetry we used *F*-tests [26]. For each trait, the levels of measurement error (ME) were smaller than the levels of asymmetry (Table 1). There were no signs for directional asymmetry except for the femur and ulna. Therefore, we obtained individual and trait specific asymmetry values after correction for DA and ME using mixed-model regression analysis. The distribution of signed asymmetries were all leptokurtic (with kurtosis ranging between 6.15 and 31.41), as often observed in FA studies, indicating inter-individual variation in developmental instability. Unsigned FA correlated significantly positively with trait size for all traits: digit 2 (*r*=0.31, *N*=174, *p*<0.0001), digit 4 (*r*=0.25, *N*=210 *p*<0.001), femur (*r*=0.28, *N*=1015, *p*<0.001), fibula (*r*=0.2, *N*=533, *p*<0.001), radius (*r*=0.24, *N*=1016, *p*<0.001), tibia (*r*=0.33, *N*=964, *p*<0.001) and ulna (*r*=0.21, *N*=992, *p*<0.001). We divided levels of unsigned FA by trait size (mean of both sides) and multiplied by 100, so that the calculated FA reflected differences in percentages of asymmetry relative to trait size. After standardizing the size-corrected unsigned FA values, we calculated average asymmetry based on the available traits. The resulting average size-corrected standardized unsigned FA values were used in further statistical analyses and will be further referred to as FA. 

**Table 1:** Overview of levels of measurement error.

**Trait**	**Real FA**	**ME-Radio**	**ME-Digitizing**	**ME-measurement**	**test for DA**	**DA p-value**
**Digit 2**	0.60	0.35	0.00	0.00	Χ^2^=0.93	0.34
**Digit 4**	1.52	1.41	0.00	0.03	Χ^2^=0.74	0.39
**Femur**	2.18	0.80	0.10	0.19	Χ^2^=39.0	<0.001
**Fibula**	2.75	1.16	0.05	0.10	Χ^2^=2.96	0.09
**Radius**	1.17	0.51	0.29	0.14	Χ^2^=2.97	0.09
**Tibia**	4.95	0.36	0.08	0.08	Χ^2^=0.00	0.99
**Ulna**	1.43	0.71	0.00	0.24	Χ^2^=4.03	0.04

Measurement errors are the result of positioning the fetus, making the radiograph (ME-Radio), digitizing the radiograph (ME-Digitizing) and measuring on a single radiograph (ME-measurement) and are relative to real FA and levels of directional asymmetry (DA) in the different limb bones of the fetuses. The variance components were multiplied by 1000.

### Associations between fluctuating asymmetry, reduced amniotic fluid volume and congenital abnormalities

We used linear models with FA as dependent variable and the amniotic fluid volume (normal volume (NV), PH, OH, AH) as independent variable. We included (log-transformed) age as continuous covariate in the model, because we observed FA to decrease with fetal age in a previous study, which used a substantial part of the same data [23]. Furthermore, when effects of uterine compression on FA are present, we expect effects to be larger over time, and thus at higher fetal age, because of fetal size and duration of compression. Age was log-transformed to assure linearity and correctly use the ANCOVA models. In addition, using age untransformed in these analyses would cause the older fetuses/infants to act as outliers [23]. If there are effects of reduced amniotic fluid volumes on FA, we expect effects to be similar for AH and OH and for PH and NV, because they represent the same biomechanical event (reduced vs. ample amniotic fluid volumes). When results are comparable we group AH and OH, and PH and NV into the two biologically meaningful groups to increase power in subsequent linear models.

To study if abnormalities in different organ systems are occurring more frequently with the different types of amniotic fluid volumes, we cross tabulated the frequencies of individuals with the different types amniotic fluid volume per affected organ system. To visualize the relationships between affected organ systems and amniotic fluid volumes we performed a correspondence analysis based on this contingency table. The correspondence analysis plots the associations between rows and columns of a contingency table in two dimensions, in such a way that rows and/or column names that occur in close proximity on the plot show a relatively high co-occurrence or association. The statistical significance of these associations between amniotic fluid volumes and affected organ systems was tested with *Χ* ^*2*^ – tests and bonferroni adjusted for multiple comparisons. 

To evaluate the direct effects of uterine compression by OH and AH on levels of FA, and to see to what extent their co-occurrence with congenital abnormalities confounds patterns in increased FA, we used linear models with FA as the dependent variable and we grouped congenital abnormalities, as found associated in the correspondence analysis, together and included them as independent factor in our model. We compared effect sizes between 1) fetuses with normal amniotic fluid volumes and no congenital abnormalities associated with reduced amniotic fluid volume, 2) fetuses with reduced amniotic fluid volumes, but without congenital abnormalities associated with AH/OH, 3) fetuses with normal amniotic fluid volumes, but with congenital abnormalities associated with reduced amniotic fluid volume and 4) fetuses with both reduced amniotic volumes and congenital abnormalities associated with those reduced volumes. All analyses were conducted in R (version 2.15.0) using the lmer function of the lme4 package for the mixed model ME and DA analysis, the ca function of the ca package for the correspondence analysis and the lm function for the ANCOVA models [27-29].

## Results

### Associations between amniotic fluid volumes and fluctuating asymmetry

**Figure 2:**
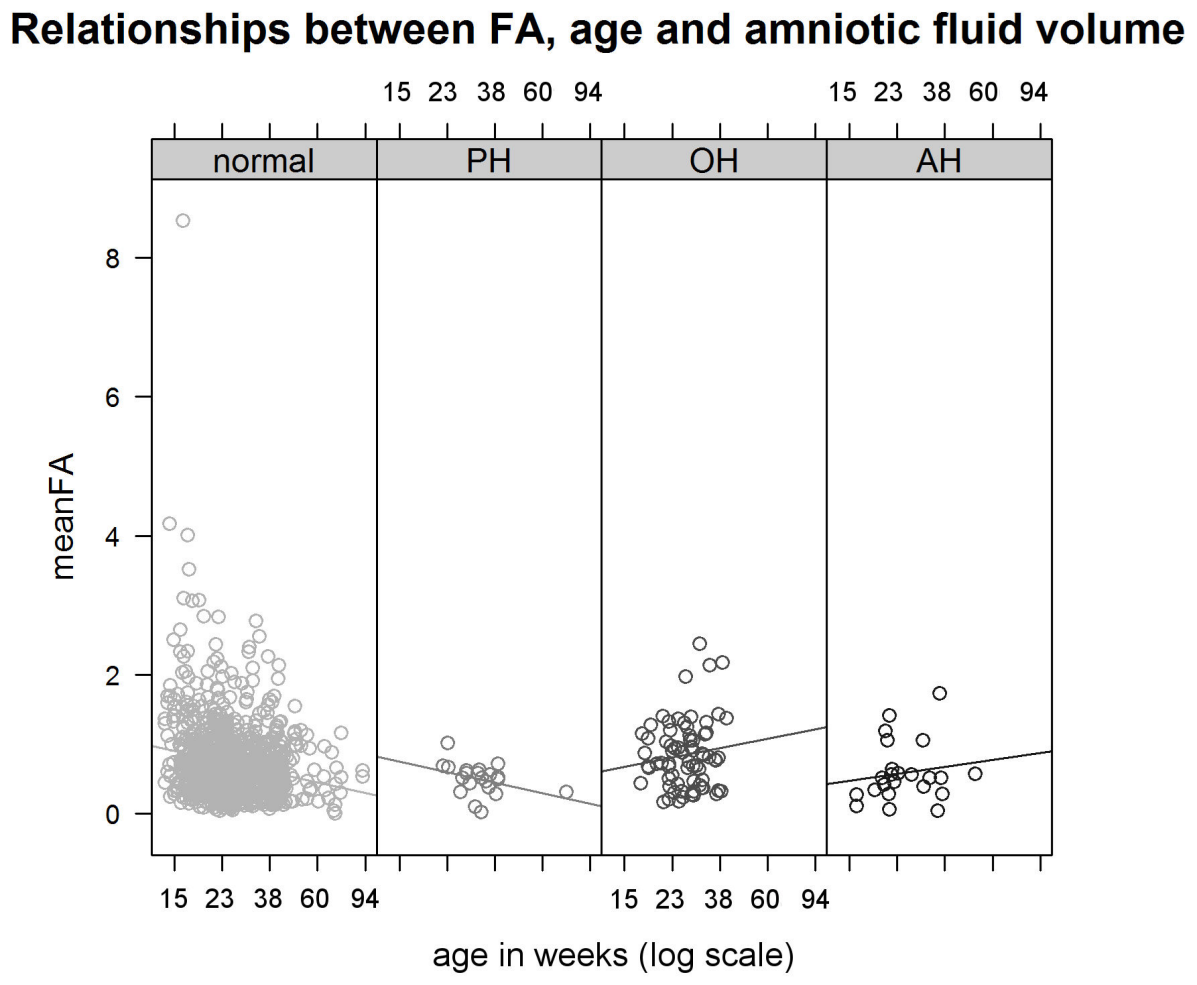
Relationships between age, FA and amniotic fluid volumes. Mean size-corrected fluctuating asymmetry decreases with age (in weeks on a log-transformed scale) for human deceased fetuses that developed in normal amniotic volumes and with polyhydramnios (PH) and increased with age for fetuses with oligohydramnios (OH) and anhydramnios (AH). Slopes of regression lines are not significantly different from each other, but are when AH/OH and normal volumes/PH are grouped together.

We compared levels of asymmetry between fetuses that developed with NV, PH, OH, or AH with (log-transformed) age added to the linear model (Figure 2). The slopes of the different groups of amniotic fluid volume differed in sign, but not significantly so (F_1,1068_ = 2.12, *P* = 0.10). For fetuses with OH or AH the slopes were positive (albeit not significantly so), while for fetuses with NV or PH, slopes were negative (slopes: AH = 0.51 + 0.93%, *P* = 0.57; OH = 0.68 + 0.73%, *P* = 0.37; NV = - 0.75 + 0.13%, *P* < 0.001; PH = -0.76 + 1.13%, *P* = 0.5). As the effects with age of ample [NV (N=928) and PH (N=21)] and reduced amniotic volumes [AH (N=23) and OH (N=70)] were similar, we grouped these fetuses in two biological meaningful groups with sufficient sample sizes. After grouping reduced fluid volumes and ample fluid volumes, the interaction between age and amniotic fluid volume was statistically significant, indicating different slopes for fetuses with reduced amniotic fluid volumes compared to fetuses with ample amniotic fluid volumes (interaction amniotic fluid volume * (log-transformed) age: F_*1,1068*_ = 2.76, *P* < 0.01). Asymmetry decreased significantly with age for fetuses with ample amniotic fluid volume (slope: -1.08 + 0.74%, F_*1,1068*_ = 6.9, *P* < 0.001) as seen in [23], and increased with age for fetuses with deficiencies in amniotic fluid, but not significantly so (slope: 0.98 + 0.73%, F_*1,1068*_ = 1.3, *P* = 0.17). To compare FA between AH/OH and PH/NV with the different direction of slopes, we estimated effects at the average age in our study (27.8 weeks): fetuses with AH/OH showed significantly higher asymmetry than fetuses with NV/PH (main effect estimates: NV/PH = 0.71 + 0.02%, AH/OH = 0.78 + 0.06%, F_*1,1068*_ = 1.97, *P* < 0.05). 

### Associations between amniotic fluid volumes and congenital abnormalities

**Figure 3:**
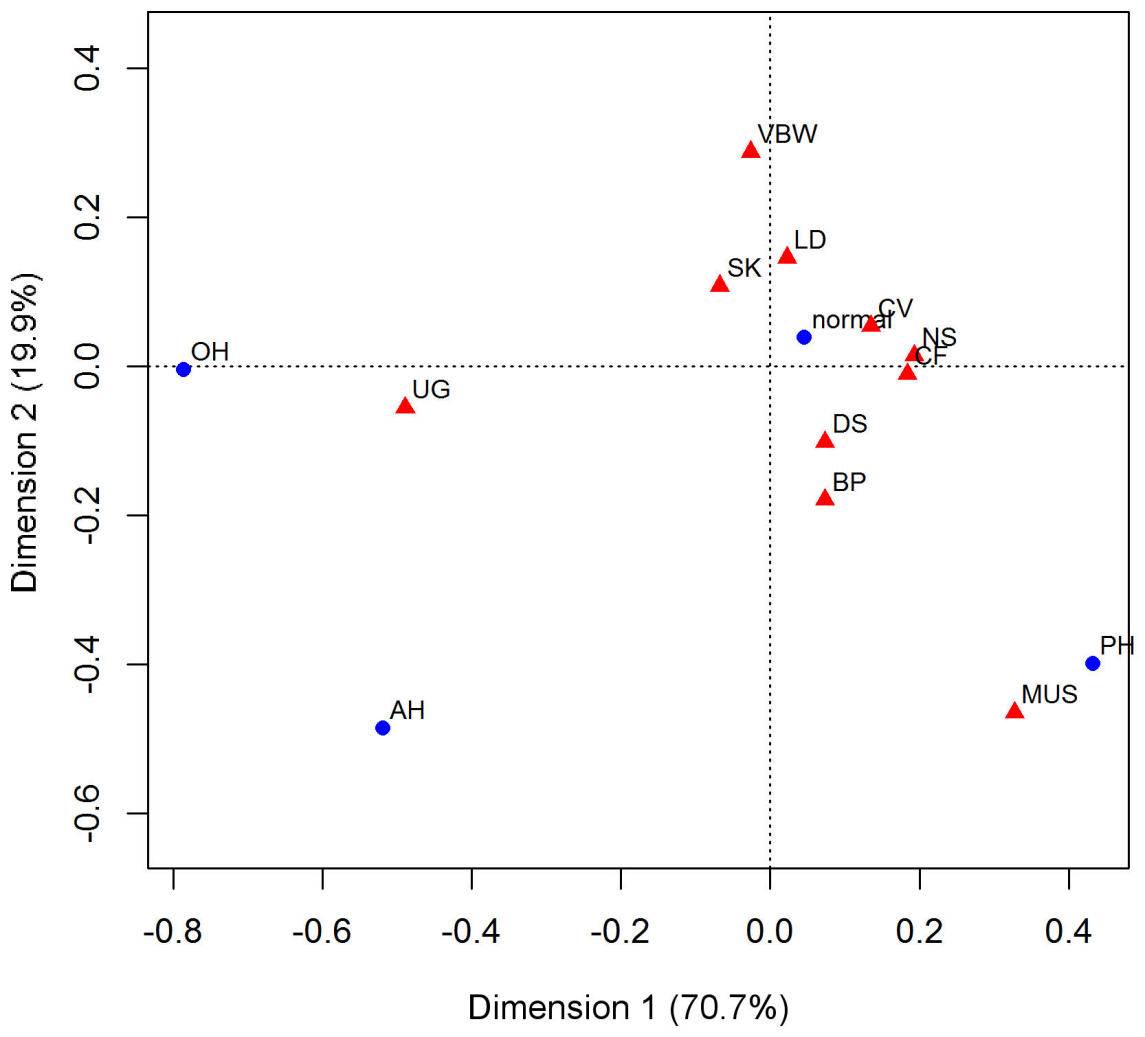
Correspondence analysis between different congenital abnormalities and amniotic fluid volumes. The correspondence analysis visualizes the contingency between the different types of congenital abnormalities and amniotic fluid volumes. The closer groups are located to each other, the stronger the correlation between the groups is. The horizontal-axis accounts for 70.7% of the variation and the vertical-axis for 19.9%. The significance of relationships between types of amniotic fluid volumes and congenital abnormalities is calculated using chi-squared tests. Oligohydramnios (OH) and anhydramnios (AH) associate significantly with urogenital defects (UG) and polyhydramnion (PH) with muscular system malformations (MUS), but not significantly after bonferroni adjustment. Normal amniotic fluid volumes associate with nervous system defects, but not significantly after bonferroni adjustment. No associations are found between cardiovascular defects (CV), skeletal defects (SK), craniofacial defects (CF), limb development abnormalities (LD), digestive system defects (DS), and ventral body wall defects (VBW) and the different types of amniotic fluid volume.

We examined the associations between congenital abnormalities in the different organ systems and the different amniotic fluid volumes (NV (N=606), AH (N=19), OH (N=42), and PH (N=35)) in a correspondence analysis (Figure 3). The first dimension (horizontal-axis) accounted for 70.7% of the variation and the second dimension (vertical-axis) for 19.9%. The correspondence analysis map of Figure 3 shows that both OH and AH correspond with urogenital problems in the lower left quadrant, i.e. a contingency between OH/AH and UG exists and these variables are thus not independent from each other. PH corresponds with muscular system malformations in the lower right quadrant. *Χ* ^*2*^ – tests based on the contingency table of the correspondence analysis confirmed that the distribution of congenital abnormalities over the different groups of amniotic fluid volume are not independent for muscular abnormalities (MUS), nervous system abnormalities (NS) and urogenital abnormalities (UG), of which only UG abnormalities remained significant after Bonferroni correction (table 2). Relationships are independent for bronchopulmonary abnormalities (BP), craniofacial abnormalities (CF), cardiovascular abnormalities (CV), digestive system abnormalities (DS), limb defects (LD), skeletal defects (SK), and ventral body wall defects (VBW) (table 2). 

**Table 2:** Overview of Χ^2^ tests on contingency between congenital abnormalities and amniotic fluid volumes.

**Organ system**	**N**	***Χ^2^***	**df**	**P**
**MUS**	19	7.60	3	< 0.05
**NS**	89	8.90	3	< 0.05
**UG**	118	32.26	3	< 0.001*
**BP**	48	3.58	3	0.31
**CF**	46	1.99	3	0.58
**CV**	148	2.95	3	0.40
**DS**	75	3.60	3	0.30
**LD**	77	0.94	3	0.82
**SK**	66	4.33	3	0.23
**VBW**	16	1.17	3	0.76

The distribution of congenital abnormalities over the different groups of amniotic fluid volume are not independent for muscular abnormalities (MUS), nervous system abnormalities (NS) and urogenital abnormalities (UG), of which only UG abnormalities remained significant after Bonferroni correction. Urogenital abnormalities associate significantly with an- and olighohydramnios (Figure 3). Relationships are independent for bronchopulmonary abnormalities (BP), craniofacial abnormalities (CF), cardiovascular abnormalities (CV), digestive system abnormalities (DS), limb defects (LD), skeletal defects (SK), and ventral body wall defects (VBW, N = number of individuals, *Χ* ^*2*^ = chi-squared value, df = degrees of freedom, P = p-value, * = significant after bonferroni adjustment).

### Associations between fluctuating asymmetry and congenital abnormalities, and the influence of amniotic fluid volumes

For the final analyses, we examined the effects of urogenital problems (i.e. the congenital abnormality which co-occurred with amniotic fluid deficiency in our dataset) and amniotic fluid volumes on FA, while adding (log-transformed) age to the model. We corrected the (log-transformed) age for the average age in order to compare effect sizes. Fetuses without urogenital abnormalities and NV showed the lowest FA at an average age (estimate: 0.70 + 0.02%). Fetuses with AH/OH, but without urogenital abnormalities had significantly higher asymmetry at an average age compared to fetuses from the former group (estimate: 0.77 + 0.07%, F_1,937_ = 2.1, *P* < 0.05). Similarly, fetuses with ample amniotic fluid volumes but with urogenital abnormalities also showed significantly higher FA than fetuses without urogenital abnormalities and with normal amniotic fluid volumes (estimate: 0.80 + 0.05%, F_1,937_ = 2.6, *P* < 0.01). As expected, fetuses with both AH/OH and urogenital problems showed the highest FA (estimate: 0.82 + 0.09%), but the combined effect of the interaction between urogenital abnormalities and reduced amniotic fluid volumes was not significant (F_1,936_ = 0.92, *P* = 0.35), indicating that the effects reduced amniotic fluid volumes and the presence of congenital abnormalities in the urogenital system were additive. Both AH/OH and UG contributed to higher limb FA and the effects of AH/OH on asymmetry did not change conclusions on associations between urogenital abnormalities and FA.

### Main results

Fluctuating asymmetry normally decreases significantly with fetal age, but we observed that fluctuating asymmetry increases with fetal age when there is not enough fluid in the amniotic cavity (albeit not significantly so). Reduced amniotic fluid volumes therefore have effects on fluctuating asymmetry, which is normally used to measure developmental instability. Urogenital abnormalities, which are described to increase fluctuating asymmetry in Van Dongen et.al. 2009 are found associated with reduced amniotic fluid volumes. We evaluated the significance of the direct effects of compression of the limbs on fluctuating asymmetry and the indirect effects of disturbed urogenital developments on FA in fetuses with urogenital abnormalities and found smaller effects sizes for the direct effects of reduced amniotic fluid volumes than for the indirect effects of disturbed development by urogenital abnormalities on FA. Correcting for the relevant direct factors allows representative testing of the associations between DI and stress.

## Discussion

### Do amniotic fluid deficiencies increase asymmetry?

Fluctuating asymmetry as measure for developmental instability and its relation to stress/quality has been widely studied. The effect sizes of associations between FA and stress/quality were found to be only weak to moderate in most reviews, but there is high heterogeneity in the literature and little is known about the sources driving this heterogeneity. To evaluate the usefulness of FA as a measure of fitness and stress, it is crucial to know which factors confound associations with FA in bilateral symmetrical traits. One aspect that has received little attention is the direct mechanical effects on development instead of indirect effects of DI (i.e., the accumulation of small developmental errors). Here, we examined the possibility that reduced amniotic fluid volume and the resulting mechanical pressure on the developing fetus may confound FA of the limb bones prenatally, similar to postnatal effects of mechanical loading due to lateralized behavior. Amniotic fluid volume rises progressively from 10-20 mL at 10 weeks of gestation, when the fetal kidneys start to develop, to 800 mL at 24 weeks, and then remains relatively constant until term. As expected, we find higher asymmetry for the limb bones if amniotic fluid volume is abnormally low. Fetuses that developed with (severe) deficiencies in amniotic fluid show a higher limb asymmetry at an average age of 27.7 weeks compared to fetuses with sufficient amniotic fluid volumes. 

This increase of subtle asymmetries was not unexpected as substantial asymmetrical development of the limbs is generally considered to be a defect due to compression [18-20,30]. Importantly, in this study we excluded all individuals with abnormal scores for limb development, such that the higher asymmetry in fetuses with amniotic fluid deficiency was not an artifact of such abnormalities, but likely a more subtle outcome of mechanical compression. Indeed, our results suggest that AH and OH are associated with an increase in limb asymmetry, although easily overlooked when not measured precisely. Limb asymmetry decreases significantly with age in fetuses with normal and elevated amniotic fluid volumes (as also shown in Van Dongen et al. [23]. A negative relationship was also found for fetuses in general in the literature [17,31]. This negative slope significantly differs from the similarly angled positive slope found for fetuses with reduced amniotic fluid volumes, although the positive estimate is not significant, presumably due to a lower number of fetuses. We expect the positive slope to be confirmed when more fetuses are investigated, because the compressive effects of OH/AH are larger in later stages of pregnancy, when the fetus takes up more place and the compression is also longer. Such an age-related effect on asymmetry would be unrelated to developmental instability.

### Which congenital abnormalities are associated with abnormal amniotic fluid volume?

Normal amniotic fluid volumes are mainly determined by fetal urination and swallowing [32]. Although its etiology often remains unknown, PH is commonly associated with abnormalities impairing swallowing (e.g., gastrointestinal obstruction, diaphragmatic hernia, fetal akinesia syndrome) [33-35]. In this study, we found PH to correspond with muscular system defects, but not with digestive system problems, only partly confirming the literature [33-35]. OH and AH can be caused by various congenital abnormalities, the majority of which involve fetal urogenital tract impairment and a reduced production or excretion of fetal urine [36,37]. Here, we found both OH and AH to correspond with urogenital abnormalities, confirming earlier results [38-40]. Altogether, the results of our correspondence analysis are in agreement with associations reported in the clinical literature.

### Are associations between FA and congenital abnormalities confounded by the effects of abnormal amniotic fluid?

The associations between amniotic fluid deficiencies and urogenital abnormalities may imply that the increased FA with urogenital problems as found by Van Dongen et al. [23] are not only the result of increased developmental instability, but also of direct biomechanical loading effects due to AH/OH. If so, the usefulness of fetal traits to study patterns in FA and to understand the factors affecting the strength of associations between FA and developmental stress might be compromised. Nonetheless, our final set of analyses showed that both AH/OH and urogenital abnormalities positively influence levels of limb asymmetry, and that when correcting for the confounding effects of AH/OH, the effects of urogenital abnormalities do not considerably change. We cannot completely exclude that the direct effects of AH/OH lead to a sequence of developmental errors truly representing DI, instead of only confounding levels of asymmetry. However, we consider this possibility unlikely, because the significant different relationship with age for fetuses with reduced amniotic fluid volumes suggests that the AH/OH effects are unrelated to DI. Thus, we conclude that the observed increases of FA with urogenital abnormalities are only to a minor extent confounded by biomechanical loading due to AH/OH and reliably indicate increases in DI. Furthermore, we conclude that fetuses are useful models to understand patterns of FA development, especially when taking into account potential confounding factors, such as those of AH/OH. We encourage further detailed study of different and more refined categories of congenital abnormalities, as this should increase our understanding of FA and symmetry development.
